# Why Should People with Lived Experience Be Included in the *DSM* Revision Process?

**DOI:** 10.1002/hast.70051

**Published:** 2026-03-16

**Authors:** Anne‐Marie Gagné‐Julien, Phoebe Friesen

**Keywords:** *DSM*, patients’ participation, lived experience, epistemic injustice, tokenism, bioethics, mental health

## Abstract

*Increasingly, scholars and advocates are recognizing the importance of including individuals with lived experience of mental health issues in the development of psychiatric research and policy. Here, we hope to contribute to discussions regarding the specific context of the* Diagnostic and Statistical Manual of Mental Disorders (DSM) *revision process. We argue that this process is not inclusive enough, but also that those who have advocated for better inclusivity have not been responsive enough to the risks reported from other inclusive mental health contexts (in research, practice, and policy). In particular, tokenism and lack of uptake of input from people with lived experience are likely to loom large in relation to* DSM *efforts at inclusivity as well. In light of this, we suggest that disentangling the reasons for inclusion can help to overcome these problems, and we offer practical recommendations for a more inclusive* DSM *revision process*.

## Article

In recent years, an increasing number of scholars and advocates have argued that people who are using or have used psychiatric services should be included at various levels of decision‐making and knowledge generation related to mental health.^1^ This participatory turn, which has been implemented in care practices, research, and professional organizations, has been motivated by many types of arguments. In this article, we focus on the arguments that have been made within the context of the *Diagnostic and Statistical Manual of Mental Disorders* (*DSM*) revision process, which is ongoing. The latest version of this manual is the *Diagnostic and Statistical Manual of Mental Disorders, Fifth Edition, Text Revision*, which was published in 2022, nine years after *DSM‐5*. Like earlier versions, *DSM‐5‐TR* was produced by the American Psychiatric Association and offers a nosology of mental disorders widely used by clinicians, researchers, and health care insurers across North America and beyond.^2^ We argue that some of the risks associated with participation have slipped under the radar of discussions about inclusivity in the *DSM* revision process. This is important given the growing dissatisfaction expressed by people with lived experience when they are involved in various forms of research and decision‐making related to mental health care. These cautionary tales are worth focusing on, as they offer lessons based on existing inclusive procedures and designs. Through these lessons, it is possible to better imagine how to develop a meaningfully inclusive *DSM* revision process while it is still under way. In a context where the APA plans to publish a sixth edition of the *DSM* (*DSM‐6*) in the coming years,^3^ a discussion on the inclusivity of the revision process is urgent.

To envision an inclusive revision process for the *DSM* that avoids some of the risks associated with participation, our argument will take place in four stages. First, we look at the *DSM‐5* revision process and at how people with lived experience have been included so far. Second, we review the different types of arguments that have been offered to justify a more inclusive review process. We propose a nonexhaustive taxonomy of three types of arguments: ethical and political arguments, epistemic arguments, and epistemic justice arguments. This taxonomy enables us to show that, despite certain changes, the *DSM‐5‐TR* does not reflect sufficient receptivity to arguments in favor of participation. Third, we make the case that, while these different arguments all justify better inclusiveness in the *DSM* revision process, they have neglected to consider the risks of tokenism and of poor uptake of input from people with firsthand experience of mental health issues, problems reported in more inclusive mental health contexts. We show how these risks represent real dangers in other, more participative contexts and argue that they are likely to apply to the *DSM* revision process as well. In the final section, we suggest that disentangling the reasons for inclusion is a promising step toward overcoming these problems, offer practical recommendations for a more inclusive *DSM‐5‐TR* revision process, and respond to potential objections that our account may face.

## Is the *DSM* Revision Process Inclusive? The New Approach of *DSM‐5*

According to John Sadler, since the publication of *DSM‐III* (1980), there have been “democratizing aspects” implemented within the *DSM* revision process.^4^ The APA has tried to diversify work groups—the committees responsible for reviewing the literature on specific sets of diagnoses and assessing the need for change—by including mental health professionals outside the field of psychiatry, such as psychologists and social workers. There has also been a willingness to remain transparent in decision‐making (by publishing drafts of modifications and their rationales, for instance) and to render these decisions open to criticisms. But, as Sadler rightly notes, this democratization has been only partial so far. For example, people with lived experience who were not also “mental health professionals” were excluded before the development of *DSM‐5*. To remedy this blind spot, the DSM Task Force made several changes during the preparation of the fifth edition of the manual.

First, public consultation was introduced during the *DSM‐5* revision process. Proposed diagnostic criteria changes were published online,^5^ and members of the public were given an opportunity to comment on the proposed changes for a two‐month period (and approximately thirteen thousand comments were received).^6^ Comments were systematically reviewed and their suggestions incorporated by work groups when deemed relevant. The public was also given the opportunity to submit petitions arguing in favor of or against certain draft changes.^7^ Consultation with “patients” was another addition to the revision process. The launch of the website was preceded by consultations with representatives of patient groups and family members, such as the National Alliance on Mental Illness (NAMI), the Depression and Bipolar Support Alliance (DNSA), and Mental Health America (MHA). This consultation aimed to target and discuss issues that directly affect patients, such as stigma, access to care, and health insurance.^8^ The DSM‐5 Task Force—the committee in charge of overseeing the overall revision process—also included a patient appointed to represent patient interests, James McNulty, former president of NAMI. In addition, patients were able to express their perspectives via online comments, and their views were in fact considered. For example, Dan J. Stein and Katharine A. Phillips reported that the working group tasked with analyzing proposed changes to the *DSM*'s discussion of obsessive‐compulsive disorders took into account patients’ expressed support of the changes in online comments.^9^ Therefore, it is fair to say that the *DSM‐5* revision process intended to be more inclusive than the processes for previous *DSM* revisions.

Despite this, there are reasons to question the efforts toward inclusion made within the *DSM‐5* revision process. While representatives from the task force have written that they “are unaware of any other area of medicine that has encouraged patient and family participation to the degree that we have attempted to do here with DSM‐V,”^10^ it is not clear that the task force's initiatives were very radical. For instance, with a few exceptions, it is not possible to know whether the work groups gave serious consideration to the comments received online in relation to the proposed modifications. Nor is it possible to know what proportion of comments came from patients versus other stakeholders or members of the public, or to access these comments. This lack of transparency about what comments were submitted online and how and whether they were considered has been decried by many.^11^ Perhaps most importantly, the single patient representative included on the task force, McNulty, is arguably not well poised to “represent the interests of mental health consumers.”^12^ NAMI, of which he was president, has long been the subject of intense criticism for its conflicts of interest and failure to adequately represent patient views.^13^ A whistleblower who worked at the biopharmaceutical company Pfizer claimed in 2009 that the organization was paid to promote the off‐label use of an antipsychotic in children. What's more, McNulty, during his presidency, was reported to have been receiving personal payments for talks he gave promoting such use.^14^ This context should give us pause before applauding the DSM Task Force for its inclusive measures.

It is also worth exploring what the main motivations were to include “patients” in the *DSM‐5* revision process. The official argument of the APA goes like this: “[P]atients, families, lawyers, consumer organizations, and advocacy groups have all participated in revising *DSM‐5* by providing feedback on the mental disorders described in this volume. *Their monitoring of the descriptions and explanatory text is essential to improve understanding, reduce stigma, and advance the treatment and eventual cures for these conditions*.”^15^ Inclusion was also justified because of “social dimensions” associated with diagnoses and the correlated “unique concerns and questions related to issues that directly affect patients, such as social stigma, the impact of potentially pejorative diagnostic terminology (e.g., *schizophrenia*) and descriptors (e.g., *addiction*), access to care, forensic issues (e.g., competency hearings, disability cases, discrimination lawsuits), and insurance coverage.”^16^ So there seems to be a recognition that the perspectives of people with lived experience are relevant to the review process and that such people can contribute both ethically (such as by identifying stigmatizing language) and epistemically (such as by contributing to the advancement of treatment).^17^ However, the fact that patients were not included in the work groups, which oversaw the assessment of evidence and recommended modifications for a specific set of diagnoses (such as mood disorders) indicates the limitations of the DSM Task Force's commitment to the democratic ideal. The work groups were primarily composed of psychiatrists but also included other mental health professionals. This was justified on the basis that the “patient‐subjective data” associated with patients’ perspectives conflicted with the APA's desire to establish the *DSM* as adhering to a scientific and objective process.^18^ Given this wide recognition of the ethical and epistemic reasons for inclusion and the narrow implementation of such inclusion, there appears to be a lack of clarity, and even a built‐in contradiction, about what people with lived experience are expected to contribute to the process.

## Arguments for Inclusivity and *DSM‐5‐TR*

Some might think that this superficial form of inclusivity might be the right approach for the *DSM*, as, ultimately, there would be no good‐enough reasons for inclusion of people with the lived experience of using or having used psychiatric services. For instance, Robert Spitzer wrote a response to a call for inclusion in *DSM‐5* in which he described participatory efforts as “politically correct nonsense.”^19^ In response to this intuition and to strengthen our claim that the new *DSM‐5‐TR* has not been inclusive enough, we illustrate a variety of reasons that have been invoked in recent years to push for more inclusion of people with lived experience within the *DSM* revision process. We divide the arguments into three types—ethical and political arguments, epistemic arguments, and epistemic justice arguments—and critique the revision process in the light of this taxonomy.

***Ethical and political arguments***. The first type of argument states that there is a moral obligation to include people with lived experience in the *DSM*. Building on a democratic ideal framework, Sadler and Bill Fulford observe that, “at a minimum, social justice suggests that stakeholders who have the most to gain or lose should be included in the processes that affect them.”^20^ Because in many respects the *DSM* diagnostic categories impact public policies, they touch on public interests. As a result, key stakeholders or constituencies (groups of people who have an interest in or who will be impacted by decisions)—here, patients—should be represented in the decision‐making processes related to these categories.

The inclusion of people with lived experience has also been defended using the principles of beneficence and autonomy. Rebecca Johnson and colleagues have argued that one main goal of a diagnostic category is to benefit patients by, for instance, providing a framework to identify and alleviate suffering.^21^ Inclusion of people with lived experience, because they have firsthand experience of how a diagnostic change can benefit or harm them, could provide valuable input on how beneficence is understood and achieved. Moreover, the authors have proposed that inclusion could ensure that affected communities would grant consent to anticipated social effects of *DSM* revisions, meeting the bioethical norm of autonomy as embodied in informed consent practices. Similarly, Douglas Porter argues that autonomy means “self‐determination of the institutional norms that affect us.”^22^ Since the categories embedded in the *DSM* have a huge influence on public life, the manual can be seen as an institutional norm. To respect patients’ autonomy, inclusion mechanisms should be put in place. The logic of this was captured in the words of a member of Anoiksisis, a Dutch patient group of service users who have been diagnosed with schizophrenia or have experienced psychosis. In response to decisions being made by the DSM‐5 Psychotic Disorders Work Group, Bill George noted, “It is after all *our* experiences that the psychiatrists are talking about and legislating over.”^23^

***Epistemic arguments***. Most arguments made in favor of including people with lived experience in the *DSM* revision process focus on epistemic reasons for inclusion. One approach takes as its starting point that scientific objectivity can have social meaning.^24^ Proponents of this approach put forward that, given the uncertainty surrounding psychiatric classification and the influence of values on the development of the manual, patient input is necessary for improving the manual. This is true for producing better knowledge of psychiatric categories,^25^ the definition of “mental disorder,”^26^ and how the notion of “dysfunction” is described.^27^ What these authors show is that, if people with lived experience have different perspectives on psychiatric diagnoses and concepts than mental health specialists have, they may then be able to identify problematic assumptions embedded in the *DSM* text and categories (such as ableist views of normality or well‐being).

A related epistemic argument is grounded in the framework of feminist standpoint theory.^28^ One of us (Phoebe Friesen) and Jordan Goldstein have provided reasons to believe that the socially subordinated position of psychiatric‐service users can grant them a potential epistemic advantage when it comes to producing knowledge related to psychiatry, in that they often have better awareness of the way power relations shape knowledge.^29^ The argument suggests that people who have firsthand experience of having been labeled with a psychiatric diagnosis occupy a particular social location; if they engage in activities of critical reflection (through, for example, consciousness‐raising groups, support groups, or research), then they are likely to be especially able to bring critical insight to research or knowledge projects. One example Friesen and Goldstein give involves the pathologization of mental distress and differences. Many self‐advocacy groups have challenged the assumption that such distress and differences should always be understood as “disorders,” “deficits,” or “dysfunctions”—a view promoted by the *DSM*.^30^

***Epistemic injustice arguments***. A third kind of argument in favor of inclusivity is tied to the framework of epistemic injustice, bringing together epistemic and ethical reasons for inclusiveness. Epistemic injustice involves wrongs committed against individuals or groups when they try to produce or communicate knowledge, and the injustice is ultimately caused by negative identity stereotypes associated with marginalized social groups to which they belong.^31^ In the context of the *DSM*, it has been generally argued that, because there are good epistemic reasons to consider patients’ testimonies in the *DSM* revision process (as described above), when these testimonies are not actively sought, the DSM Task Force is committing a testimonial injustice. For instance, Anke Bueter argues that the APA is committing this kind of wrong because “patients are excluded from participation in virtue of belonging to the group of laypeople, who are taken to have nothing competent or relevant to contribute to scientific practices.”^32^ This exclusion leads to two detrimental effects: the diagnostic criteria may not correspond to the lived experience of those diagnosed, and the range of values contained in the diagnostic criteria will be constrained and will tend to represent the interests of mental health professionals only. But there is also a moral harm, in that the reason patients are excluded is grounded in prejudice, rather than merely an absence of knowledge.^33^

Integrating patients in the *DSM* revision process can also help develop what Bueter calls “public epistemic trustworthiness.”^34^ Because of the pervasive influence of values in psychiatric nosology and because patients have reasons to distrust that the classification is elaborated in their best interests (given, for example, the influence of the pharmaceutical industry and the history of pathologizing differences such as the diagnosis of homosexuality in the *DSM I* and *II*), integrating patients can help to build trust. Through their inclusion, patients could act as gatekeepers in the review process, ensuring that their interests are represented and therefore giving other patients more reason to believe that the *DSM* diagnostic categories represent them.

**DSM‐5‐TR *and inclusivity***. As demonstrated, a range of arguments have been put forward in favor of better integrating people with lived experience into the *DSM* revision process. These arguments were developed in parallel with the revision of the *DSM‐5*, or shortly after its publication. Since the publication of *DSM‐5* in 2013, however, one significant change has been implemented within the *DSM* revision process. In particular, a continuous improvement model has been adopted. This means that revisions are made on an ongoing basis according to new evidence (including the risk of harm of a diagnosis) and changes are published online as they are recommended by the review committees (which are similar to the work groups for *DSM‐5*) and approved by the steering committee (which is similar to the task force for the *DSM‐5*). Then, as with the *DSM‐5* revision process, the proposed changes are made open to comments by the public, and based on these comments, the steering committee makes modifications and submits the final version of change to the APA Assembly and Board of Trustees for approval. In 2022, the revised version, *DSM‐5‐TR*, was published in hard copy and included revised texts and updated changes.^35^

While this is an important step toward democratizing the process of revising the *DSM*, little has changed with regards to the inclusion of those with lived experience. There is no patient perspective represented on the review committee, and only one patient representative sits on the steering committee. While anyone can request a change in the *DSM* at any moment, such requests “must be accompanied by supportive information in a structured format, including the reasons for the change, the magnitude of change, data documenting improvements in validity across a range of validators, evidence of reliability and clinical utility, and consideration of current or potential deleterious consequences associated with the proposed change.”^36^ This online comment option is characterized by the same shortcomings as the previous *DSM‐5* revision process—a lack of power shifting, inadequate representation of people with firsthand experience of receiving psychiatric services, a mere advisory approach, and no transparency about how public comments are, or should, be handled. This means that, despite all the arguments set out above, the *DSM* has taken only the tiniest step toward more meaningful participation of people with lived experience in the revision process. A call for greater inclusiveness is therefore still urgent. That said, the iterative nature of the *DSM‐5‐TR* and the APA's explicit commitment to include relevant stakeholders in the process of publishing *DSM‐6* ^37^ appear to present an opportunity for better inclusion in the future.

## Tokenism and Failures of Uptake in Participatory Psychiatry

While those engaged in discussions of inclusivity and the *DSM* revision process all agree that more inclusion is necessary, there has been little discussion of how such inclusion ought to take place. In this section, we hope to contribute to this important conversation by drawing attention to reported difficulties associated with projects of inclusion in other domains of psychiatry.^38^ In particular, we examine common and interrelated concerns about tokenism and failures of uptake, both of which arise frequently in the context of participatory settings.

Tokenism, a superficial form of involvement, is widely reported by those who have been involved in participatory research initiatives related to psychiatry. One peer researcher, describing their experience of involvement, notes that the motivation for their inclusion was that the lead researcher wanted to say they “have this visibly trans person on my team who is, you know, leading the project, even though I was a tiny person on it, like I had nothing to do with it [*laughs*]. So I think a lot of it was optics really.”^39^ This sense of being used, commodified, or exploited is far too common in the experiences of service users involved in mental health projects.^40^ As Sarah Carr has put it, “Many of us do not want to be used as specialist data collectors on someone else's project or as token lucrative commodities in funding bids, but want to change research cultures and knowledge production to make it broader and more inclusive.”^41^

Relatedly, many who have been involved in participatory processes related to mental health research report a lack of clear benefit or uptake within projects they have been involved in. For example, one peer researcher explains of their experience, “I'm left with a bit of bitterness about that project to be honest, because I did so much work on that project .… I don't know what has happened with that work .… [W]hat's changed? I see nothing.”^42^ Such concerns have been raised in the context of participatory efforts related to mental health care as well. In a report titled “Participation—Why Bother? The Views of Black and Minority Ethnic Mental Health Service Users on Participation in the NHS [National Health Service] in Bradford,” Heather Blakey reports that doubts circulate related to whether researchers have a “genuine commitment to change; getting involved is seen to take a lot of effort and energy for very uncertain results.”^43^

These issues related to tokenism and failures of uptake are sometimes related to a lack of clarity surrounding what service users are expected to contribute to a project or how their contributions might be used. Survivor‐researcher^44^ Jasna Russo reports, “My personal experiences of collaborative research projects have always given me fewer and fewer reasons to want to collaborate, and more and more reasons to initiate user/survivor‐controlled projects. The amount of ‘room’ for user researchers in the project varies, but there is often no clarity about why we are there and what exactly is expected from us.”^45^ Particularly when inclusion is required by funders and not something committed to by the researchers, issues related to role confusion are all too common.^46^

Another part of the problem is that there is often little room for authentic participation because decisions about how the research will look, how care will be altered, or how policies might be written have all largely been made in advance of inclusion. As Jayasree Kalathil describes, “The hierarchical power structures of the mental health professional‐patient relationship are replicated in many user involvement spaces. Merely having some service users around the table is sometimes seen as equality enough. Often, agendas have already been set and decisions made.”^47^ Sometimes this is due to structural features (for example, funding has already been secured, and so decisions related to research methods and outputs have already been made), but other times, those leading the participatory process are not actually open to shifting power in any substantial way.

Unsurprisingly, participants often feel unheard and disempowered as a result of these experiences.^48^ Kalathil notes, “Participating as a token representative in these situations can aggravate feelings of disempowerment already felt on account of discriminatory experiences based on one's racial identity, mental health status and position in society.”^49^ Interestingly, however, Lori Ross and colleagues, who conducted interviews with thirty‐four peer researchers about their experiences, report that these researchers often made calculated decisions in which harmful experiences during participation were balanced with the potential for impact: “[P]articipants [the peer researchers] often made strategic decisions to permit themselves to be tokenized, out of the expectation of promised benefits to their communities.”^50^

As many of these reports suggest, inclusion often creates more harm than good, exacerbates experiences of stigmatization, or discourages people with lived experience from taking part in these collaborative research projects,^51^ particularly in the context of marked power relationships.^52^ However, taking these risks of participatory efforts into account can help us develop informed recommendations for better inclusion in the *DSM‐5‐TR*.

## Rethinking the *DSM* Revision Process

***Clarifying reasons***. Tokenism and failures of uptake are significant issues related to the meaningful inclusion of people with lived experience in mental health care and research. Considering how widespread they are in these contexts, there is good reason to think that similar issues are likely to arise within inclusion efforts related to the *DSM* revision process. As previously argued, these issues are especially likely to occur if there is a lack of clarity about why inclusion ought to take place. There are various reasons that patient populations might be invited to participate in research or decision‐making, and in each case, the patients involved and the form of involvement ought to reflect these reasons. For example, sometimes, patients are invited to be coresearchers for instrumental reasons (to help with recruitment or to identify meaningful outcome measures in a clinical trial, for instance). In such cases, patients selected to participate in the research will be chosen as a result of their experiential expertise. In other cases, patient involvement makes a project more legitimate (as in the development of a biobank that will be using large amounts of patient data). In these cases, representativeness will matter more than expertise, given that the few patients involved are to speak on behalf of many.^53^

This suggests that achieving a better and more inclusive process for the *DSM*—which would minimize the risks of tokenism and failure of uptake and, ultimately, of harming those being included—will require getting clear on the reasons for inclusion. As demonstrated above, there is currently both contradiction and a lack of clarity in the reasons offered by the APA to justify inclusion of people with lived experience in the *DSM* revision process. It is therefore reasonable to expect the same kind of problems within the *DSM* revision process as witnessed in other participatory mental health contexts. However, the taxonomy of arguments presented above can serve as a useful basis for thinking about the reasons underlying the goal of greater inclusiveness in this revision process, with the aim of clarifying how inclusion ought to look. Here, we offer three lessons stemming from the reasons offered, followed by a discussion of practical recommendations.

First, clarifying the reasons for inclusion will necessarily involve looking to both epistemic and ethical justifications. As shown above, there are good reasons for inclusion that are founded on moral principles like justice and autonomy, and there are also good reasons for inclusion found in an examination of what people with lived experience can uniquely contribute to the *DSM* process in terms of knowledge production. While epistemic injustice arguments help to bridge this gap, they tend to still focus on epistemic reasons for inclusion while documenting both moral and epistemic harms of exclusion.^54^ As a result, looking across epistemic and ethical reasons may help clarify how precisely the APA ought to take up the challenge of appropriate inclusion of people with lived experience in the *DSM* revision process.

Second, the taxonomy of reasons for inclusion can help with anticipating risks of tokenism and failure of uptake associated with greater inclusiveness in the *DSM*. Indeed, it is likely that inclusion for epistemic reasons will fall into different traps than will inclusion for moral ones. For example, a focus on epistemic reasons related to what people with lived experience can contribute in terms of knowledge generation and improvement (by, say, being able to identify or criticize problematic background assumptions in the *DSM*) might lead to a failure to adequately share power or address issues of justice. This can result in tokenism, given that those included can be seen as merely instrumental informants. Similarly, an exclusive focus on ethical and political reasons might lead to a failure to adequately consider the potential epistemic contribution of people with lived experience to knowledge production. Inclusion for ethical or political reasons could lead to better power distribution in the process but could also lead to failures of uptake, in that there may be confusion about what particular role and contributions those included are expected to make. It may be understood that people with lived experience deserve to participate in the process because the *DSM* revisions will influence them in the future but not if, what, and how they can contribute in terms of knowledge production.

***Recommendations***. These lessons directly inform our recommendations related to how the *DSM* revision process ought to take up the goal of inclusion of people with lived experience. While these recommendations are neither definitive nor exhaustive, we hope they will provide a starting point for discussions and developments in this space. First, taking up both ethical and epistemic reasons for involvement will mean making changes to the *DSM* revision process that recognize both the rights of those impacted by the manual to be involved in its development and the expertise that this community can offer. Furthermore, taking up reasons that are epistemic, ethical, and related to epistemic injustice will help to ensure that risks related to tokenism and failures of uptake can be minimized within inclusion processes.

Given this, the first modification we suggest aims to address both ethical and epistemic reasons for inclusion. We recommend the creation of what we call a “people with lived experience committee” (hereafter “PLE committee”) to inform the ongoing revisions of the *DSM*. This committee, which will be entirely composed of people with lived experience, will participate in reviewing online feedback, act as a liaison with service‐user communities and patient‐led organizations, and facilitate accessible avenues for feedback and discussions of nosology. This way, the PLE committee will contribute to both moral and epistemic ideals of inclusion (in that people with lived experience will be allowed to have a say in the nosological changes that will affect them and to contribute to knowledge development associated with concepts and categories of the *DSM*).

Our second recommendation is that all existing *DSM* review committees (work groups) be modified to include at least two representatives with relevant lived experience of the condition(s) under consideration to participate in the revision of diagnostic criteria. The need for inclusion in the review committees is more salient when we focus on epistemic advantages people with lived experience bring to assessing evidence and making decisions regarding diagnoses. These epistemic advantages involve the recognition that one's lived experience may offer a unique form of expertise and that expertise may be relevant to decisions related to how that experience ought to be understood and classified. This change is likely to lead to the inclusion of service users who are directly affected by the condition(s) or symptoms being considered and who are in community with others who are also affected. Such experts by experience are best placed to foresee potential gaps, assumptions, or promising developments that come along with nosological changes.

Lessons related to moral and epistemic injustice reasons for inclusion will be important to keep in mind across these changes. As discussed above, it is well recognized that participatory mental health research often fails to truly address ethical and political arguments and that, instead, power remains only in the hands of those leading the research.^55^ This risk will be present in any inclusive *DSM* revision processes as well. To minimize it, the decision‐making authority of those representing patients must be clearly determined and communicated in advance. For example, will the PLE committee have veto power over decisions made by review committees or have the ability to pause the implementation of a change so that further consultation with the affected community or communities can take place? Clarity regarding roles and expertise will be especially important in the review committees to avoid tokenism and failures of uptake. In other committees that have sought to incorporate “community” perspectives (such as institutional review boards), assumptions related to expertise and confusion related to what community representatives are meant to contribute has led to the dismissal of community‐level contributions and to self‐silencing on the part of community members.^56^ To avoid this, the role, expertise, and expected contribution of people with lived experience on each review committee should be specified in advance and understood by all members of the committee. For example, will those with lived experience be asked to review proposed changes with a particular eye to issues related to access, stigma, language, and so forth? Providing clarity on these aspects of inclusion will help avoid common risks faced in such processes.

Essentially, when attentive to both epistemic and ethical reasons, the *DSM* revision process is more likely to offer clarity about the roles those included ought to play, the contributions they are expected to make, and the populations they are intended to represent, as well as how their contributions will be taken up, thereby limiting the risks of tokenism and failures of uptake. Therefore, the new *DSM* revision process will be able to be more inclusive and to avoid some of the core pitfalls of other participatory endeavors.

## Objections

Some might worry that one issue we have not substantially addressed so far is the question of representation of a group so diverse and complicated as people with lived experience and, accordingly, of how to select the “right” people for the PLE committee and for the review committees.^57^ First, we acknowledge that representation is a substantial issue in any efforts related to inclusion and that our proposal is not going to solve this issue entirely. There simply is no version of a PLE committee that perfectly represents all people with lived experience, and adding two representatives to each review committee will not be sufficient either. That said, we want to emphasize that our account allows progress in answering these complicated questions in that clarification of reasons for inclusion can also inform representation. For example, with regards to our first recommendation about forming a PLE committee to inform revisions, attention to ethical reasons for participation may lead to the selection of representatives who have already been appointed by service‐user communities in some way (for example, those involved in patient‐led organizations, survivor research, and peer‐led initiatives). In contrast, attention to epistemic reasons may lead to the inclusion of people with lived experience with a variety of backgrounds (in terms of social belonging, medical experiences, and visions of mental health), enhancing the chance of being able to reveal problematic background assumptions embedded in the *DSM* text and categories. Taking both of these reasons into account when considering questions of representation will be important, we propose. Moreover, these two kinds of reasons can help explain why we should be careful as to the potential conflicts of interest that can accompany such positions, as in the case of McNulty, and could prevent the achievement of both moral and epistemic ideals. While there is substantial discussion of representation in the literature related to the inclusion of service users,^58^ our account makes explicit the need to consider representation along both ethical and epistemic lines. In this regard, it offers a fruitful starting point by specifying the kind of questions that need to be asked to better address the issue of representation for inclusive *DSM* revision practices. Moreover, in line with the arguments presented above, this type of question should be answered not solely by scientific experts but also by people with firsthand experience.

Another objection that one might raise in response to our recommendations is whether there are important limits related to the knowledge and insight that people with lived experience can bring to the *DSM* revision process. This issue may seem salient when considering the inclusion of such individuals in the review committees responsible for evaluating scientific literature on different diagnostic categories. For example, Sam Fellowes raises important worries about the kinds of expertise that service users may lack. Fellowes notes that constructing a nosological system like the *DSM* is likely to require expertise related to how theoretical virtues (such as simplicity, coverage, embeddedness in a scientific network, identified causal mechanisms, and accuracy) can be best achieved in that system, and other experts, namely psychiatric researchers and clinicians, may be more likely than service users to have such expertise. As Fellowes points out, achieving these theoretical virtues might require “understanding that goes far beyond lived experience, such as knowledge of how symptoms cluster in many different people, knowledge of many different psychiatric diagnoses and knowledge of areas of science other than psychiatric diagnoses.”^59^ However, we do not propose to replace the expertise of other members of the *DSM* working groups with only that of people with lived experience but merely to complement it. This means that expertise related to these virtues will remain mostly in the hands of scientific experts, but will benefit from the essential, additional viewpoint of those who understand mental health conditions from the inside. What's more, based on the epistemic justifications presented in our taxonomy, it is arguable that experiential expertise may contribute to achieving these theoretical virtues in scientific processes like *DSM* revision, in partnership with scientific experts. For example, an understanding of appropriate symptom coverage, which Fellowes notes is part of constructing an adequate nosological system of mental disorders, ought to be informed by the values and experiences of those experiencing symptoms; this can ensure the inclusion of symptoms that are best classified as pathological and the exclusion of those that are not (for example, hearing distressing voices versus hearing helpful voices).^60^ Thus, supplementing scientific expertise with experiential expertise does not undermine the scientific nature of the *DSM* revision process. Rather, it recognizes that certain scientific decisions can benefit from the input of people with lived experience.
